# Imaging-defined residual risk after left atrial appendage occlusion: from device surveillance to antithrombotic and reintervention decision considerations

**DOI:** 10.3389/fcvm.2026.1881673

**Published:** 2026-07-09

**Authors:** Yuan Li, Yujian Liu

**Affiliations:** 1 Department of Ultrasound, Zigong Fourth People's Hospital, Zigong, Sichuan, China; 2 Department of Radiology, Zigong First People's Hospital, Zigong, Sichuan, China

**Keywords:** antithrombotic therapy, cardiac computed tomography angiography, device-related thrombus, hypoattenuated thickening, left atrial appendage occlusion, peri-device leak, residual risk, transesophageal echocardiography

## Abstract

**Background:**

Left atrial appendage occlusion (LAAO) is increasingly used for stroke prevention in selected patients with nonvalvular atrial fibrillation, particularly when long-term oral anticoagulation is unsuitable, poorly tolerated, or clinically undesirable. Although contemporary trials and registries have demonstrated favorable procedural success and long-term outcomes, post-implantation imaging findings continue to create uncertainty during follow-up. These findings include device-related thrombus (DRT), hypoattenuated thickening (HAT), peri-device leak (PDL), residual left atrial appendage patency, and device-appendage mismatch.

**Purpose:**

This review reframes post-LAAO imaging findings as imaging-defined residual risk markers and proposes an evidence-informed framework that explicitly links management considerations to their principal supporting evidence sources.

**Key content:**

DRT is uncommon but clinically relevant, with event rates of ischemic stroke or systemic embolism of approximately 6.3 per 100 patient-years in patients with DRT vs. 1.7 per 100 patient-years in those without DRT. HAT is a heterogeneous imaging phenotype ranging from expected healing to thrombus-like surface abnormality. CCTA detects residual appendage patency and PDL more frequently than TEE, but greater sensitivity does not automatically confer clinical actionability. This review organizes post-LAAO findings into three domains: device surface abnormality, residual communication, and device-appendage geometry.

**Conclusion:**

Post-LAAO imaging should move beyond binary device surveillance toward risk-weighted interpretation. Most management implications remain supported by expert consensus, observational evidence, and registry data rather than prospective trials. Outcome-linked imaging thresholds and prospective validation are required before imaging-defined residual risk can be incorporated into clinical decision pathways.

## 1 Introduction: from device success to residual risk

LAAO has become an established nonpharmacological strategy for stroke prevention in selected patients with nonvalvular atrial fibrillation. Its rationale rests on the dominant role of the left atrial appendage as a source of thrombus formation and on the need for alternatives to indefinite oral anticoagulation in patients with high bleeding risk, prior bleeding, poor tolerance, or other barriers to long-term anticoagulant therapy (1–11). Two recent randomized trials have strengthened this rationale. PRAGUE-17 demonstrated the noninferiority of LAAO vs. direct oral anticoagulants, with lower nonprocedural clinically relevant bleeding (3). In OPTION, LAAO after atrial fibrillation ablation reduced nonprocedural bleeding while remaining noninferior for death, stroke, or systemic embolism at 36 months (4). These trials do not imply that successful implantation eliminates all residual thromboembolic risk. Post-LAAO assessment is therefore shifting from confirming procedural success to characterizing residual risk after implantation.

Relevant post-implantation findings can be grouped into three domains: device-surface abnormalities, residual communication, and device-appendage geometry. These findings differ substantially in biological meaning and clinical actionability. DRT is clinically relevant, HAT spans expected healing and thrombus-like abnormalities, and CCTA frequently detects residual communication whose prognostic significance remains uncertain (12–16). The central challenge is to distinguish clinically meaningful residual risk from benign healing, limited anatomic communication, or imaging over-detection.

Current guidelines and position statements acknowledge persistent uncertainty in surveillance imaging, adjunctive antithrombotic therapy, and the management of DRT and PDL, while emphasizing the need for standardized acquisition and interpretation (5, 16). Interpretation should also remain device-specific because occluder design influences expected post-implantation appearance and residual-risk mechanisms (17–19).

Several high-quality reviews have summarized multimodality imaging for LAAO. Cepas-Guillen et al. (2025) organized post-LAAO imaging assessment by temporal phase, from immediate post-procedural evaluation to long-term follow-up, and addressed the complementary roles of TEE and CCTA across these phases (20). Jain et al. (2025) provided a comprehensive overview of multimodality imaging for pre-procedural planning, intra-procedural guidance, and post-procedural surveillance (21). The present review differs from these contributions in three respects. First, it organizes post-LAAO findings into a three-domain residual-risk framework—device surface abnormality, residual communication, and device-appendage geometry—that is oriented toward management translation rather than descriptive summary. Second, every clinical consideration is explicitly linked to its principal supporting evidence source, including randomized trial protocols, registry data, cohort studies, case series, and expert consensus. Third, device-appendage geometry is elevated from a secondary technical descriptor to an independent interpretive domain considered alongside surface abnormality and residual communication, while recognizing that the three domains do not have equal evidentiary strength or clinical actionability. The novelty lies primarily in the integrative, management-oriented organization of existing evidence rather than in the introduction of entirely new imaging entities. The framework is provisional and intended to support evidence-aware interpretation and individualized multidisciplinary decision-making rather than function as a validated treatment algorithm.

## 2 Post-LAAO imaging surveillance: modalities, timing, and interpretation framework

Post-LAAO imaging surveillance has traditionally confirmed device position, excluded DRT, assessed PDL, and guided antithrombotic de-escalation. In early Watchman studies, follow-up TEE at approximately 45 days determined whether oral anticoagulation could be discontinued when no DRT was present and residual peri-device flow was absent or within the accepted threshold (1, 22, 23). Contemporary practice is more heterogeneous owing to evolving devices, broader patient selection, alternative antithrombotic regimens, and increasing use of CCTA (5–11, 15, 16, 24–32).

TEE remains the historical reference modality and is indispensable in selected clinical scenarios. It offers real-time assessment of device stability, mobile thrombus, peri-device flow by color Doppler, and adjacent cardiac structures. Established protocols include multiplane interrogation at 0°, 45°, 90°, and 135°, with color Doppler used to identify and measure peri-device flow (11, 12, 22, 24, 33–37). Limitations include semi-invasiveness, need for sedation in many patients, operator dependence, acoustic shadowing, and incomplete characterization of distal appendage opacification beyond the occluder.

Contemporary echocardiographic techniques extend standard two-dimensional TEE. Three-dimensional (3D) TEE may improve spatial localization of PDL, delineate its circumferential extent, and clarify its relationship to device geometry (11). Contrast-enhanced TEE has been proposed as an adjunct for selected equivocal cases to improve detection of small peri-device flow jets and help discriminate artifact from true residual communication, although dedicated post-LAAO validation remains limited (11). These techniques are most valuable when standard Doppler findings are equivocal. The current literature should not be read as favoring CCTA over TEE in all scenarios; rather, the two modalities are complementary tools that interrogate overlapping but distinct aspects of device performance.

CCTA has emerged as an increasingly important complementary modality. Its strengths include high spatial resolution, three-dimensional visualization of the device-appendage relationship, direct assessment of device position and compression, and detection of contrast opacification distal to the device. CCTA is also sensitive for HAT and residual appendage patency, including patterns that may not produce a visible color Doppler jet on TEE (15, 16, 24, 38–42). A 2025 meta-analysis confirmed higher CCTA sensitivity for any residual communication, but no significant difference for large PDL (>5 mm) or DRT detection (15). This supports CCTA as a surveillance option but also creates a familiar interpretive problem: CCTA may reveal more abnormalities than are currently proven to be clinically actionable.

Intracardiac echocardiography (ICE) has become increasingly relevant for procedural guidance. Registry and observational data suggest that ICE and TEE guidance can achieve comparable procedural success in selected LAAO settings (34–36, 43). Its current role, however, is mainly intraprocedural, because postprocedural ICE is invasive and not practical for routine outpatient surveillance.

CCTA may identify residual appendage patency without a corresponding Doppler-visible PDL. Such CCTA-only findings should not automatically be interpreted as high-risk leak or closure failure because their mechanisms and clinical significance remain heterogeneous (15, 16).

Surveillance timing remains unsettled. Common time points include 45 days, 3 months, 6 months, and 12 months, although institutional protocols and device-specific guidance vary. The 2025 SCAI/HRS guideline recognizes persistent practice variation in imaging, adjunctive antithrombotic therapy, and management of DRT and PDL (5).

**Figure 1:**
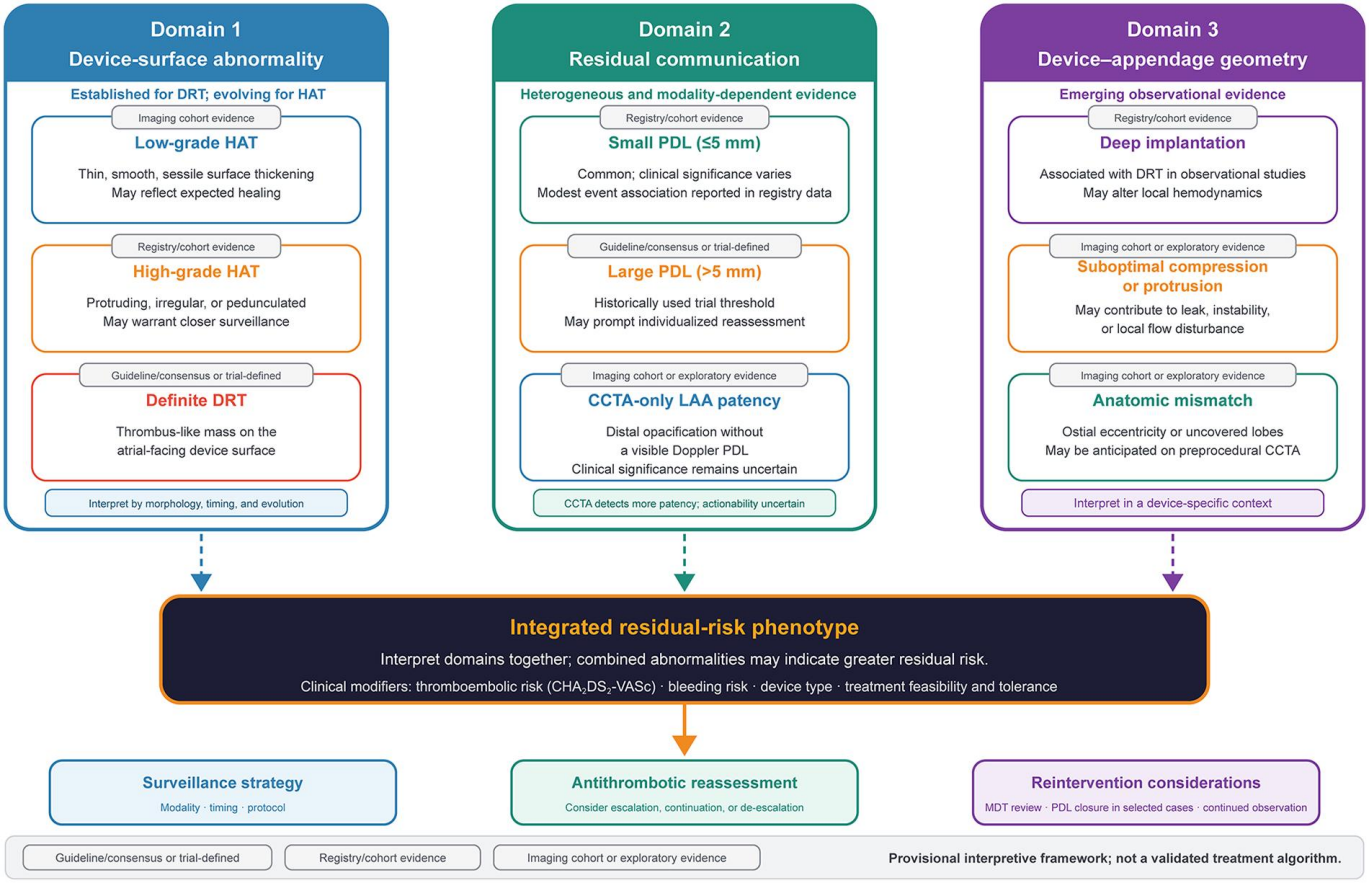
Three-domain framework for imaging-defined residual risk after LAAO. Post-LAAO imaging findings are organized into three interrelated domains. These domains should be interpreted together and integrated with thromboembolic risk, bleeding risk, device type, and treatment feasibility and tolerance. CCTA, cardiac computed tomography angiography; DRT, device-related thrombus; HAT, hypoattenuated thickening; LAA, left atrial appendage; LAAO, left atrial appendage occlusion; MDT, multidisciplinary team; OAC, oral anticoagulation; PDL, peri-device leak; TEE, transesophageal echocardiography.

Post-LAAO imaging should therefore be organized around a structured three-domain framework (Figure 1). Device surface abnormality asks whether material on the atrial-facing device surface may represent expected healing, HAT, thrombus-like abnormality, or definite DRT. Residual communication asks whether the appendage remains anatomically or functionally connected to the left atrium. Device-appendage geometry asks whether implantation depth, compression, protrusion, uncovered lobes, exposed components, or device-anatomy mismatch create conditions for stasis, leak, or thrombogenicity.

The domains are most useful when read together. A small PDL in a well-seated device may not carry the same implication as a similar leak accompanied by deep implantation, high-grade HAT, or prior embolic events. Surveillance imaging should define a risk phenotype that can be integrated with thromboembolic risk, bleeding risk, device type, and treatment tolerance.

## 3 Device surface abnormalities: HAT, DRT, and delayed endothelialization

Device surface abnormality is the imaging domain most directly linked to potential thromboembolic risk. It encompasses a spectrum from expected device healing to HAT, high-grade thrombus-like surface abnormality, and definite DRT. Their appearances, timing, and biological substrates overlap, which makes graded interpretation more useful than a single binary label (Table 1).

**Table 1:** Imaging-defined residual risk phenotypes after LAAO: definitions, quantitative data, and imaging findings.

Domain	Phenotype	Definition and Key Imaging Features	Typical Modality	Key Quantitative Data
Device Surface Abnormality	Low-grade HAT	Flat/sessile, 1–3 mm, smooth contour, low suspicion of thrombosis; device-specific criteria apply	CCTA (may be occult on TEE)	Prevalence ∼24% at ∼4 months (14) No stroke association: HR 0.75, *p* = 0.62 (14) Complete resolution in small serial-imaging subset (6/6) (42)
	High-grade HAT	>3 mm, pedunculated/irregular, high suspicion of thrombosis; in Watchman FLX classification: protruding sessile or pedunculated (41)	CCTA ± TEE correlate	Prevalence ∼5% at ∼4 months (14) Stroke HR 4.6 (95% CI 1.5–14.0) (14) Inter-observer *κ* ≥ 0.78 (48)
	Definite DRT	Mobile or fixed echogenic/attenuated mass on device surface; independent motion variable	TEE and/or CCTA	Incidence 1.7%–3.9% (12, 13) Ischemic stroke/SE: 6.28 vs 1.65/100 pt-yr (13)
Residual Communication	PDL (Doppler-visible)	Peri-device color Doppler jet; contrast passage along device margin on CCTA; measurable gap	TEE (primary); CCTA	≤5 mm in 25.8% of NCDR patients (33) RCT protocol threshold: 5 mm (1, 22, 23)
	CCTA-only residual patency	Distal LAA contrast opacification without visible peri-device gap or TEE correlate	CCTA (may be occult on TEE)	Detected more frequently by CCTA vs TEE (15) Clinical significance uncertain
	Large persistent PDL	Continuous wide channel >5 mm; Doppler-visible or CCTA-confirmed	TEE + CCTA	≤0.7% in NCDR Registry (33) May warrant continued OAC (33)
Device-Appendage Geometry	Adverse geometry	Deep implantation, inadequate compression, protrusion, uncovered lobes, device-appendage mismatch	CCTA (multiplanar); TEE	Deep implantation associated with DRT risk (12, 44) Modifiable procedural predictor

CCTA, cardiac computed tomography angiography; DRT, device-related thrombus; HAT, hypoattenuated thickening; HR, hazard ratio; LAA, left atrial appendage; NCDR, National Cardiovascular Data Registry; OAC, oral anticoagulation; PDL, peri-device leak; SE, systemic embolism; TEE, transesophageal echocardiography. Evidence-source summaries are qualitative and should not be interpreted as formal guideline evidence grades. Device-specific classification criteria may differ across platforms.

DRT has traditionally been regarded as the most clinically important surface complication after LAAO. Reported incidence varies because of differences in device type, antithrombotic strategy, imaging modality, timing, and diagnostic criteria. In pooled PROTECT AF and PREVAIL data, DRT occurred in approximately 3.7% of patients; in Amulet IDE, approximately 3.9%; in PINNACLE FLX, approximately 1.7%; and across pooled registry and meta-analysis data, approximately 3.8% (6, 12, 13, 17–19). DRT is uncommon but not rare enough to ignore, especially because it may occur beyond the early post-implantation period.

In the pivotal analysis by Dukkipati et al. of pooled WATCHMAN trial cohorts, the rate of ischemic stroke or systemic embolism was 6.28 per 100 patient-years in patients with DRT vs. 1.65 per 100 patient-years in those without DRT, corresponding to an adjusted rate ratio of 3.22 (95% CI 1.58–6.55; *p* = 0.001) (13). Importantly, 17 of 65 patients with DRT experienced an ischemic stroke or systemic embolism during follow-up, but most post-LAAO embolic events occurred in patients without documented DRT (13). This underscores that DRT is an important but incomplete marker of residual thromboembolic risk. Meta-analyses generally corroborate an approximately two- to four-fold increase in ischemic events with DRT, although effect estimates vary across datasets, and most patients with DRT do not experience subsequent embolic events (12, 13, 44, 45).

Several predictors have been repeatedly identified. Patient-related factors include permanent atrial fibrillation, prior stroke or transient ischemic attack, renal dysfunction, reduced left ventricular function, and spontaneous echo contrast (12). Deep implantation is among the most consistent modifiable procedural predictors, probably because it may leave more exposed device surface, alter local flow, or create low-flow recesses near the device-appendage interface (12). Device-specific mechanisms may differ; in Amulet IDE, DRT timing and location differed between Amulet and Watchman platforms (12, 18, 19).

HAT expands the range of CCTA-visible surface abnormalities. Unlike definite DRT, HAT is an imaging phenotype rather than a pathological diagnosis. Its substrate may include endothelialization, fibrin deposition, organized thrombus, fresh thrombus, inflammatory tissue, or mixed healing response (12, 14, 16, 41, 46, 47). CCTA makes HAT more visible because small or sessile abnormalities may be difficult to detect with TEE.

The most systematic quantitative characterization of HAT comes from Iriart et al., who prospectively evaluated 412 patients who underwent follow-up CCTA at a mean of 4.2 months after LAAO (14). Using a classification based on thickness, morphology, and suspicion of thrombosis, HAT was absent in 71.1% of patients. Low-grade HAT—defined as flat or sessile thickening measuring 1–3 mm with low suspicion of thrombosis—was present in 23.8% of patients. High-grade HAT—defined as thickening exceeding 3 mm, pedunculated morphology, or irregular contour with high suspicion of thrombosis—was identified in 5.1% (14). During a median follow-up of 17 months, stroke occurred in 5.8% of the cohort. High-grade HAT was independently associated with ischemic stroke (hazard ratio 4.57; 95% CI 1.49–13.97; *p* = 0.008), whereas low-grade HAT showed no significant association (hazard ratio 0.75; *p* = 0.62) (14). High-grade HAT was also strongly associated with antithrombotic therapy discontinuation (odds ratio 9.5; 95% CI 3.1–29.1; *p* < 0.001) (14). The accompanying editorial by Choe reinforced these findings and highlighted the importance of standardized grading (46). It should be noted that the Iriart classification was developed across device types and that device-specific grading systems—such as the Watchman FLX classification described below—may use different criteria. High-grade HAT is generally characterized by thickness >3 mm and/or protruding, irregular, or pedunculated morphology, depending on the grading system used.

For the Watchman FLX platform, Kramer, Korsholm, and colleagues performed a retrospective analysis of 244 patients with 8-week post-implantation CCTA, supplemented by canine histology correlation (41). HAT was present in 64% of scans and was classified into four device-specific categories: subfabric hypoattenuation (24%), flat sessile HAT (32%), protruding sessile HAT (7%), and pedunculated HAT (1%). Subfabric and flat sessile patterns correlated with benign device healing and endothelialization, whereas protruding sessile and pedunculated HAT were considered potential DRT (41). A subsequent multicenter reproducibility study of this classification demonstrated substantial inter-observer agreement for overall HAT assessment (kappa ≥0.61), with the highest agreement for identification of high-grade HAT (kappa ≥0.78) (48).

The temporal evolution of HAT is directly relevant to surveillance interval recommendations but remains incompletely characterized. Available serial CCTA data suggest that low-grade HAT may regress, remain stable, or become more apparent over time, depending on device type and imaging interval. In a small serial-imaging subset of six patients with low-grade HAT at 8 weeks, all showed complete resolution on repeat CCTA at 12 months (42). Among patients with high-grade HAT, approximately half showed regression after a mean of 84 days with antithrombotic management, although some persisted (42). Long-term CCTA follow-up data over a median of 5.8 years after Amulet implantation showed that low-grade HAT prevalence increased from 12% at 2 months to 40% beyond 4 years; the investigators interpreted this as potentially reflecting progressive device incorporation or endothelialization, although histopathologic confirmation was unavailable (49). No high-grade HAT was observed at late follow-up in that cohort. These data underscore that HAT is a dynamic imaging phenotype and that the timing of surveillance imaging influences both detection rate and clinical interpretation.

The most useful interpretive model is therefore a continuum from expected healing and endothelialization through low-grade and high-grade HAT to definite DRT. Low-grade HAT, particularly when thin, smooth, sessile, and continuous with the left atrial wall, may reflect the healing spectrum. Protruding, irregular, enlarging, or pedunculated abnormalities are more concerning and may overlap with DRT (12, 14, 41, 46, 47).

Delayed or incomplete endothelialization provides the biological bridge between HAT and DRT. The traditional rationale for short-term post-LAAO antithrombotic therapy is that the device surface becomes endothelialized within an early healing window. Human explant and imaging observations suggest that endothelialization may be incomplete or heterogeneous beyond the first 45–90 days after implantation (12).

Imaging quality is critical. On CCTA, beam-hardening artifact, contrast mixing, cardiac motion, inadequate left atrial opacification, and lack of delayed imaging can mimic or obscure surface abnormalities (12, 16, 38, 42). On TEE, confidence improves when suspected thrombus is seen in multiple planes, is in contact with the device, is not explained by artifact or normal healing, and has morphology inconsistent with expected incorporation (12). Independent motion is not always present and should not be an absolute diagnostic requirement (12).

The clinical implications of surface abnormalities depend on morphological grade, temporal evolution, associated residual communication, and patient-level thromboembolic and bleeding risk. Definite DRT has the clearest management implications, whereas low-grade HAT generally remains within the healing spectrum and high-grade HAT warrants greater concern. Detailed antithrombotic and surveillance considerations are discussed in Section 6. HAT grade alone has not been prospectively validated as a treatment-triggering criterion (5, 12–14).

Surface abnormalities and residual communication frequently coexist. These domains are mechanistically interdependent and should be interpreted as a unified risk phenotype rather than evaluated in isolation.

## 4 Residual communication: PDL, residual LAA patency, and clinical significance

The terminology used for residual communication is summarized in Table 2.

**Table 2:** Terminology of post-LAAO residual communication.

Term	Definition	Primary Detection Method
Peri-device leak (PDL)	Residual channel around the device margin; appearing as peri-device color Doppler flow on TEE or contrast passage along the device circumference on CCTA	TEE (color Doppler); CCTA (contrast along device margin)
Residual LAA patency	Persistent contrast opacification distal to the occluder, with or without a visible peri-device gap; a broader and more sensitive CCTA finding than PDL	CCTA (primary); may be occult on TEE
Trans-fabric leak	Contrast passage through the device fabric rather than around the device margin; distinct from a peri-device gap	CCTA (contrast through fabric plane)
Microleak	Subclinical-scale residual communication below the detection threshold of standard color Doppler or without a measurable dimension	Research CCTA protocols; contrast-enhanced TEE

These entities are not mutually exclusive. PDL may occur without residual LAA patency when the leak is hemodynamically trivial, whereas residual LAA patency may be present without a visible PDL because of trans-fabric flow or limited spatial resolution of the imaging modality.

The historical interpretation of PDL was strongly shaped by early Watchman protocols. In PROTECT AF and PREVAIL, TEE at approximately 45 days assessed device thrombus and residual flow before antithrombotic de-escalation, and residual peri-device flow ≤5 mm was considered acceptable for discontinuation of warfarin (1, 6, 22, 23, 25). This threshold is better understood as a pragmatic, trial-based cutoff than as a validated biological risk boundary. Across percutaneous LAAO studies, definitions of complete closure have ranged from near-absence of flow to thresholds of 3 mm or 5 mm (50).

Early evidence suggested that residual peri-device flow was common but not clearly associated with adverse outcomes. In the PROTECT AF substudy, peri-device flow was present in approximately one third of patients at 12 months, yet increasing leak size was not associated with a higher rate of the composite efficacy endpoint (22). The low event rate, however, limited confidence. Absence of a statistically significant association should not be misread as proof that PDL is benign.

Surgical LAA exclusion trials provide background evidence that appendage exclusion can contribute to stroke prevention in selected surgical patients (51, 52). However, residual communication after surgical ligation has been associated with incomplete exclusion and thrombus persistence in historical imaging studies (53–55).

More contemporary registry data have challenged the assumption that small leaks are clinically irrelevant. In the NCDR LAAO Registry analysis of 51,333 patients, small leaks (>0 to ≤5 mm) occurred in 25.8% of patients, whereas large leaks (>5 mm) occurred in only 0.7% (33). Compared with no leak, small leaks were associated with modest but statistically significant increases in stroke, transient ischemic attack, systemic embolization, major bleeding, and major adverse events (33). Large leaks were not significantly associated with adverse events, but these patients were more frequently maintained on anticoagulation, making causal interpretation difficult (33). Subsequent reviews and imaging studies have further emphasized that the clinical significance of residual leak and CCTA-detected LAA patency remains heterogeneous and modality-dependent (24, 39, 40).

CCTA has refined and complicated the evaluation of residual communication. The 2025 meta-analysis comparing CCTA and TEE showed that CCTA detected residual leak or LAA patency and any PDL more frequently than TEE, but there was no significant difference for large PDL (>5 mm) or DRT (15). CCTA therefore adds most by identifying smaller or anatomically subtle residual communications, including residual LAA patency without visible PDL. The clinical significance of these CCTA-only findings remains uncertain (15).

Residual communication can be interpreted across three levels. A morphologic leak is defined by visible residual contrast opacification, a peri-device gap, or a channel identifiable by CCTA or color Doppler. A hemodynamic leak implies that the communication is sufficient to permit meaningful flow, typically inferred from Doppler-visible jets or robust CCTA contrast passage. A clinical-risk leak—the most demanding category and the one with the weakest evidence base—requires that the finding carries sufficient probability of thromboembolism or recurrent events to alter management. The clinical actionability, principal evidence base, and major limitations associated with the main imaging-defined residual-risk phenotypes are summarized in Table 3.

**Table 3:** Clinical actionability and evidence basis of imaging-defined residual risk phenotypes.

Phenotype	Clinical Actionability	Key Evidence Basis	Principal Limitations and Evidence Gaps
Definite DRT	Generally prompts consideration of temporary or resumed OAC when bleeding risk permits	Observational/registry data (12, 13); event rates 6.28 vs 1.65/100 pt-yr (13); guideline consensus (5)	No randomized trial of DRT management strategies; most patients with DRT do not experience embolic events
High-grade HAT	May justify closer surveillance and individualized antithrombotic reassessment	Cohort data: stroke HR 4.6 (14); inter-observer *κ* ≥ 0.78 (48); expert consensus	Strength of association derived from single prospective cohort; HAT grade alone not prospectively validated as treatment-triggering criterion
Low-grade HAT	Generally insufficient to justify antithrombotic escalation in isolation	Cohort data: no stroke association, HR 0.75, *p* = 0.62 (14); likely represents healing (41, 42)	Long-term natural history incompletely characterized; device-specific grading criteria still evolving
Large PDL (>5 mm)	May warrant continued OAC or consideration of percutaneous closure in selected patients	RCT protocol precedent (1, 22, 23); NCDR Registry data (33); PDL closure case series (56)	No randomized trial of PDL closure vs continued surveillance; NCDR large-leak group confounded by higher OAC use
Small PDL (≤5 mm)	Not dismissible as benign; integrate with clinical risk and device geometry	NCDR Registry: modest increase in thromboembolic and bleeding events (33); observational cohorts	Prognostic significance confounded by antithrombotic therapy; no imaging-triggered management trial
CCTA-only residual patency	Clinical significance uncertain; generally insufficient to justify treatment escalation in isolation	Meta-analysis: higher CCTA sensitivity but uncertain actionability (15); expert consensus	Mechanisms include true leak and non-leak causes (contrast equilibration, incomplete endothelialization); prognostic significance unknown
Adverse geometry	May modify interpretation of surface and communication findings; integrate with other domains	Observational data linking deep implantation to DRT (12, 44, 57, 58); registry associations	No validated geometric risk score; computational flow modeling remains investigational

CCTA, cardiac computed tomography angiography; DRT, device-related thrombus; HAT, hypoattenuated thickening; HR, hazard ratio; LAA, left atrial appendage; OAC, oral anticoagulation; PDL, peri-device leak; pt-yr, patient-years.

Clinical implications are intended to support individualized interpretation and multidisciplinary discussion.

Device type modifies interpretation. In PINNACLE FLX, most patients had no detectable PDL at 1 year, and all residual leaks measured ≤3 mm (6, 17). In Amulet IDE, the dual-seal Amulet occluder achieved higher closure rates than Watchman, although early procedure-related complications were higher (6, 18, 19). These data support device-specific interpretation rather than a universal leak-risk rule.

Reintervention evidence is reviewed in Section 6; residual communication should otherwise be interpreted according to size, imaging modality, associated surface findings, and clinical context.

## 5 Device-appendage geometry: malposition, compression, protrusion, and anatomic mismatch

Device-appendage geometry is the third major domain of imaging-defined residual risk. It governs the interaction between the device surface, appendage wall, and left atrial cavity, determining the distribution of residual flow channels and zones of local blood stasis. The LAA has substantial anatomical variability in ostial shape, depth, lobar structure, and landing-zone geometry (22, 50). A device may appear acceptable in fluoroscopic or echocardiographic views yet still leave eccentric gaps, uncovered lobes, or incomplete wall apposition. CCTA is particularly well suited to characterize these mismatches through multiplanar and three-dimensional reconstructions of the full device circumference (16).

Preprocedural CCTA planning connects directly with postprocedural residual risk. A recent systematic review and meta-analysis supports the additive value of CCTA-guided preprocedural planning (59), while prior expert recommendations and anatomical studies have established the role of CCTA in detailed appendage characterization and procedural guidance (38, 60–63). Some postprocedural leaks and geometric risk features are predictable from preprocedural anatomy, supporting the radiology-centered principle that preprocedural and postprocedural imaging should be interpreted as a continuum.

Implantation depth is one of the most important geometric variables. Deep implantation has been repeatedly associated with DRT risk and is among the most consistent modifiable procedural predictors (12, 44, 57, 58). Mechanistically, deep implantation may expose a larger portion of device surface to the left atrial cavity, create low-flow recesses, or expose structural components that contribute to thrombogenicity (12, 44, 57, 58).

Device compression is also critical. Insufficient compression may predispose to instability or PDL, whereas excessive compression may distort device architecture or produce nonuniform surface geometry. Expected compression differs across device platforms. In PINNACLE FLX, unsuccessful implantation was sometimes related to inadequate final compression and/or incomplete LAA seal (17).

Protrusion into the left atrium may generate local flow disturbance on the atrial-facing surface, increase exposure of thrombogenic material, or interfere with adjacent structures. Protrusion does not automatically imply risk, but it becomes more relevant when accompanied by HAT, DRT, Doppler-visible PDL, or prior embolic events (12, 16).

### 5.1 Device-specific geometric and imaging considerations

Post-LAAO imaging findings should be interpreted with explicit reference to the implanted device, because design differences influence the expected appearance, the mechanisms of thrombosis and residual communication, and the thresholds for clinical concern.

Watchman (Boston Scientific). The original Watchman device uses a single-seal plug mechanism with a polyethylene terephthalate (PET) fabric covering. It relies on radial force and distal anchoring tines for stability. Incomplete wall apposition around an elliptical ostium may result in eccentric PDL. Deep implantation exposes a larger portion of the threaded hub and fabric surface to the left atrial cavity, which has been associated with DRT risk (12, 44).

Watchman FLX (Boston Scientific). The next-generation FLX device was designed with improved recapturability, a closed distal end, broader size compatibility, and enhanced conformability to the appendage anatomy (17). The reduced device depth and rounded profile decrease the likelihood of deep protrusion, and DRT occurred in 1.7% of patients in PINNACLE FLX (17). The device-specific HAT classification is described in Section 3. From a geometric perspective, particular attention should be paid to the proximal screw-hub cove, where subfabric hypoattenuation may mimic surface thrombus; cove-height measurements also show comparatively lower inter-observer reproducibility (ICC <0.75) (48).

Amplatzer Amulet (Abbott). The Amulet occluder uses a dual-seal lobe-disc design: a distal lobe anchored within the appendage and a proximal disc that covers the ostium from the left atrial side (18, 19). The dual-seal mechanism achieves higher complete closure rates than single-seal devices. In Amulet IDE, DRT developed more frequently on the disc surface, and some DRT occurred later in follow-up (18, 19). The disc surface is a larger thrombogenic interface exposed to left atrial flow. On CCTA, HAT involving the disc should be assessed using the same graded morphological criteria applied to other platforms, recognizing that disc geometry differs fundamentally from a plug-based device (47). Five-year data suggest that device-related factors, including DRT or PDL ≥3 mm, may precede adverse events in some device groups (19).

These device-specific considerations have practical reporting implications. A flat low-attenuation region near the screw hub cove of a Watchman FLX should not be interpreted in the same way as a protruding mass on the disc of an Amulet device. Post-LAAO imaging reports should specify the device type, generation, position, compression, protrusion, implantation depth, and leak pathway if identifiable (16, 50).

Device-appendage geometry also bridges imaging and future risk prediction. Computational flow modeling suggests that device type, implantation position, local flow velocity, wall shear stress, and stagnation may influence DRT formation (12, 57, 58, 64–68). These approaches remain investigational but reinforce the principle that post-LAAO thrombosis is shaped by the local hemodynamic environment created by the interaction between device and appendage.

### 5.2 Representative multimodality imaging examples across residual-risk domains

**Figure 2:**
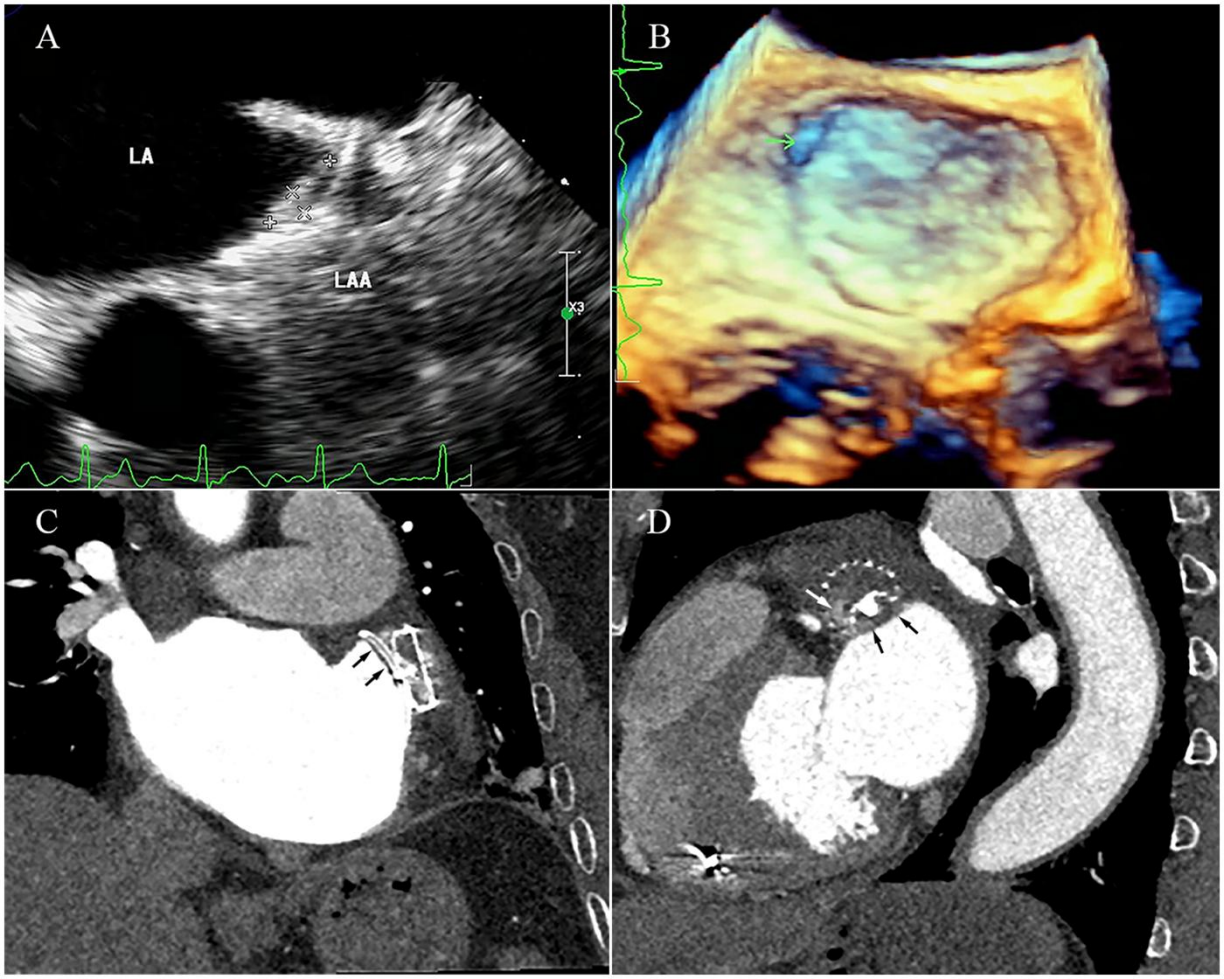
Multimodality imaging appearances of device-related thrombus and hypoattenuated thickening after left atrial appendage occlusion. **(A)** Two-dimensional transesophageal echocardiography demonstrates a mass-like echogenic lesion attached to the atrial surface of the occluder device; caliper markers delineate the lesion extent, consistent with device-related thrombus. **(B)** Three-dimensional transesophageal echocardiography further depicts the spatial relationship between the surface-attached lesion and the occluder device (green arrow). **(C)** Coronal cardiac computed tomography angiography shows a thin, smooth, mural hypoattenuated layer along the device surface (black arrows), consistent with low-grade hypoattenuated thickening. **(D)** Sagittal cardiac computed tomography angiography demonstrates a focal, thicker, protruding hypoattenuated lesion on the device surface (black arrows), consistent with high-grade hypoattenuated thickening; the associated peri-device leak is indicated by a white arrow. CCTA, cardiac computed tomography angiography; DRT, device-related thrombus; HAT, hypoattenuated thickening; LA, left atrium; LAA, left atrial appendage; PDL, peri-device leak; TEE, transesophageal echocardiography.

Figure 2 illustrates representative post-LAAO imaging findings, including device-related thrombus, low- and high-grade hypoattenuated thickening, and peri-device leak. Two-dimensional transesophageal echocardiography demonstrates a mass-like echogenic lesion attached to the atrial surface of the occluder device, consistent with device-related thrombus. Three-dimensional transesophageal echocardiography further delineates the spatial relationship between the lesion and the device surface. Cardiac computed tomography angiography illustrates the morphological distinction between low-grade and high-grade hypoattenuated thickening: low-grade hypoattenuation appears as a thin, smooth, mural layer along the device surface, whereas high-grade hypoattenuation is more focal, thicker, and protruding. The high-grade example is accompanied by a peri-device leak, highlighting the potential coexistence of surface abnormality and residual communication.

## 6 Translating imaging-defined residual risk into antithrombotic and reintervention decision considerations

**Figure 3:**
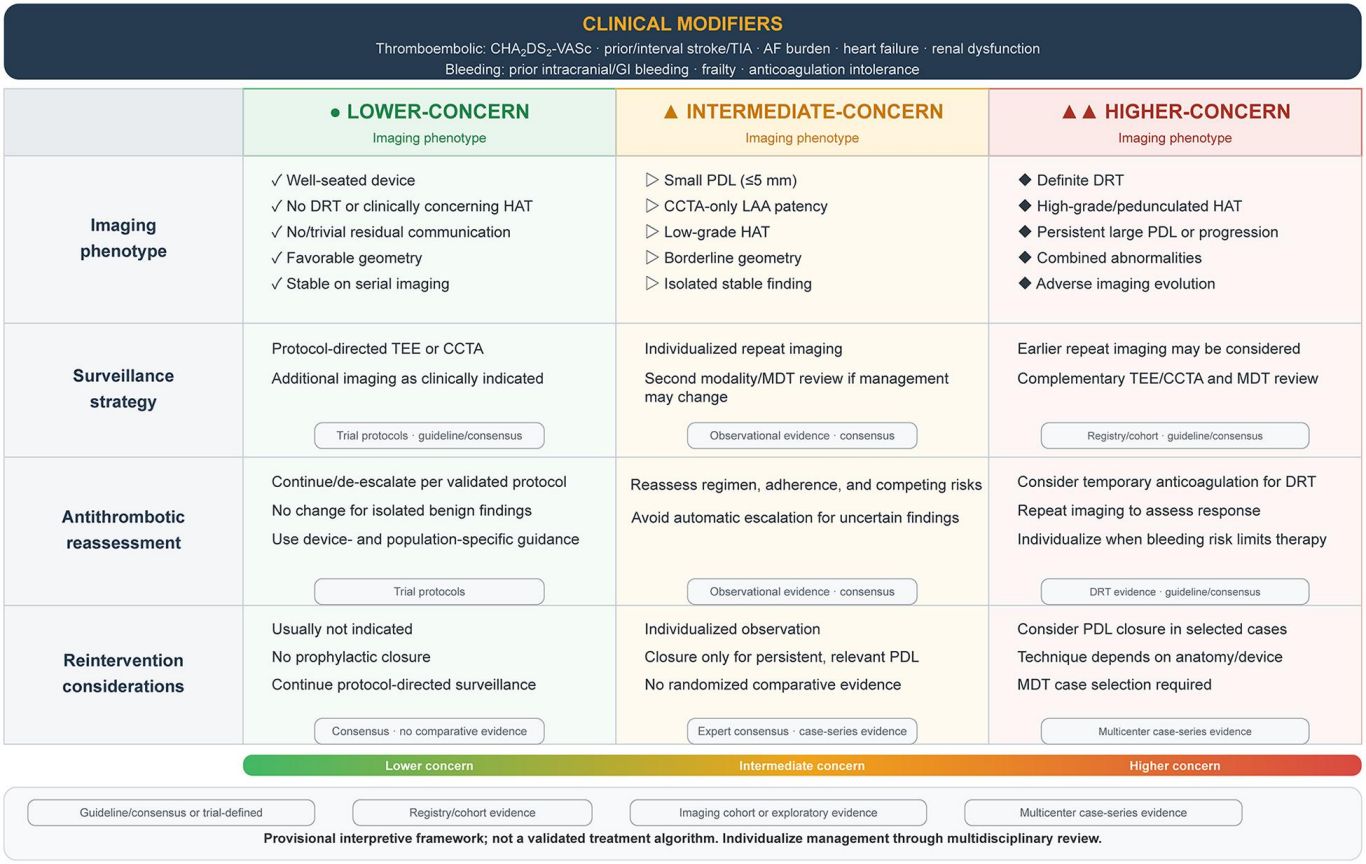
Provisional interpretive matrix for post-LAAO imaging findings. Lower-, intermediate-, and higher-concern imaging phenotypes are mapped to potential surveillance strategies, antithrombotic reassessment, and reintervention considerations. The matrix links each management consideration to its principal supporting evidence source but is not intended as a validated treatment algorithm. Clinical decisions should be individualized, particularly when imaging findings are discordant, bleeding risk is high, or evidence is uncertain. CCTA, cardiac computed tomography angiography; DRT, device-related thrombus; HAT, hypoattenuated thickening; LAA, left atrial appendage; LAAO, left atrial appendage occlusion; MDT, multidisciplinary team; PDL, peri-device leak; TEE, transesophageal echocardiography; TIA, transient ischemic attack.

The central clinical question after LAAO is not whether imaging can detect abnormalities, but whether those abnormalities should change management. At present, this question cannot be answered by a validated treatment algorithm. Evidence linking post-LAAO imaging findings to antithrombotic therapy or reintervention comes from randomized trial protocols, observational registries, expert consensus, and selected case series, rather than prospective trials that randomize patients according to imaging-defined residual risk. The framework proposed here is intended to structure evidence-aware interpretation and multidisciplinary discussion. Imaging findings should be translated into decision considerations, not rigid treatment recommendations (Figure 3).

A fundamental principle is that increased imaging sensitivity does not automatically translate into improved clinical outcomes. CCTA detects residual LAA patency and small PDL more frequently than TEE, but the prognostic significance of many CCTA-only findings remains unproven (15, 16). Treating every CCTA-only residual patency as a clinically meaningful leak could lead to unnecessary anticoagulation in precisely the population in whom bleeding risk often motivated LAAO. Distal LAA contrast opacification without a visible peri-device channel and subfabric hypoattenuation near the proximal screw-hub cove of a Watchman FLX device without a corresponding TEE abnormality are generally insufficient, in isolation, to justify management escalation. These findings warrant documentation and may influence surveillance intervals, but their independent clinical actionability has not been established by prospective outcome data.

HAT illustrates why the graded distinction matters. Low-grade, smooth, sessile HAT on CCTA without a corresponding TEE finding should not automatically be treated as DRT. Protruding, irregular, enlarging, or pedunculated HAT may reasonably be treated as a higher-risk surface phenotype, particularly when accompanied by clinical embolic events, persistent atrial fibrillation, prior stroke, or deep implantation (12, 14). Available cohort evidence supports this graded interpretation, although HAT grade has not been prospectively validated as a treatment-triggering criterion (14).

Residual communication presents a similar problem. The traditional >5-mm PDL threshold remains useful for communication and historical comparison but is not a fully validated outcome-derived threshold (22, 23, 33, 50). Small leaks are common and were historically considered of uncertain significance. NCDR LAAO Registry data showed that small leaks (>0 to ≤5 mm) were associated with modestly increased thromboembolic and bleeding events (33). This finding does not imply that every small leak requires anticoagulation or closure. Rather, small leaks should not be dismissed without considering clinical context, device geometry, and associated surface abnormalities.

Management considerations can be organized around three interacting dimensions: imaging-defined residual risk, clinical thromboembolic risk, and bleeding or treatment-tolerance risk. Imaging-defined residual risk includes definite DRT, high-grade HAT (thickness >3 mm, pedunculated or irregular morphology), large or persistent PDL, residual patency with visible communication, and adverse device geometry. Clinical thromboembolic risk includes prior stroke or transient ischemic attack, high CHA2DS2-VASc score, persistent or permanent atrial fibrillation, and hypercoagulable conditions. Bleeding and treatment-tolerance risk includes prior intracranial hemorrhage, gastrointestinal bleeding, severe anemia, frailty, falls, and inability to tolerate oral anticoagulation or dual antiplatelet therapy.

Patients with a lower-concern phenotype generally have a well-seated device, no DRT or clinically concerning HAT, no or trivial residual communication, favorable device-appendage geometry, and no concerning clinical events. Usual follow-up and antithrombotic de-escalation according to the applicable institutional or device-specific protocol may be reasonable (evidence level: trial protocol precedent and consensus practice, with limited direct outcome validation).

An intermediate-concern phenotype includes small PDL (>0 to ≤5 mm), CCTA-only residual LAA patency, low-grade HAT, borderline compression, or isolated geometric irregularity without adverse imaging evolution or thromboembolic events. Repeat imaging at an individualized interval, determined by imaging morphology, device type, temporal evolution, clinical context, and whether the result is expected to alter management, may be preferable to immediate treatment escalation (evidence level: observational imaging cohorts, registry associations, and expert consensus).

A higher-concern phenotype includes definite DRT, high-grade HAT, large persistent PDL, residual communication associated with thromboembolic events, coexisting leak and surface thrombus, or adverse geometry such as deep implantation accompanied by a surface abnormality. These patients may warrant closer surveillance, temporary anticoagulation when feasible, or consideration of percutaneous leak closure in selected cases (evidence level: DRT registry and meta-analysis data, guideline consensus, and selected PDL closure case series; treatment pathways remain unvalidated by randomized trials).

Antithrombotic therapy after LAAO remains heterogeneous. Historical Watchman protocols used warfarin plus aspirin initially, followed by dual antiplatelet therapy and then aspirin alone (1, 22, 23). The final 2-year EWOLUTION results showed favorable outcomes in a high-risk population in which many patients used antiplatelet-only or no antithrombotic therapy during follow-up (69). A large NCDR analysis of Watchman FLX recipients suggested that anticoagulation alone, without aspirin, was associated with fewer adverse events and less bleeding than anticoagulation plus aspirin or dual antiplatelet therapy, without an apparent increase in stroke or DRT (28). More antithrombotic therapy is therefore not always better, and the optimal post-LAAO regimen must balance DRT prevention against bleeding risk (26, 27, 29–32, 70).

Reintervention is less standardized. Percutaneous PDL closure may be considered in selected patients with persistent, clinically concerning leaks. In an international multicenter study of 95 patients, 104 leaks were treated with technical success of 100% and functional success of 82.7% (56). Smaller series have described endovascular coiling and radiofrequency energy for selected peri-device leaks (71, 72). These series demonstrate technical feasibility in carefully selected cases but do not support routine indications for leak closure (50).

The highest-value clinical scenarios are those with overlapping abnormalities. A small isolated PDL may be observed, but a small PDL plus high-grade HAT, prior stroke, or deep implantation may carry different implications. CCTA-only residual patency is uncertain in isolation but more concerning if accompanied by robust distal opacification, visible peri-device channel, surface thrombus, or adverse geometry. These combined phenotypes are where structured imaging interpretation can add value and where prospective studies are most needed.

In summary, imaging-defined residual risk should support individualized decision-making rather than mandate uniform treatment. Definite DRT is the clearest trigger for antithrombotic reassessment. High-grade HAT, persistent or large PDL, residual patency with visible communication, and adverse geometry are potential risk modifiers. Low-grade HAT and CCTA-only residual patency remain uncertain and are generally insufficient to justify treatment escalation in isolation. Until outcome-linked thresholds are validated, the safest framework integrates imaging phenotype, thromboembolic risk, bleeding risk, device type, and patient preference through multidisciplinary review (Table 4).

**Table 4:** Key evidence gaps and considerations for multidisciplinary review.

Residual-Risk Phenotype	MDT Considerations	Key Evidence Gaps	Research Priorities
Lower-concern phenotype (well-seated device, no DRT, no high-grade HAT, no or trivial leak)	Routine follow-up per institutional or device-specific protocol; antithrombotic de-escalation as clinically appropriate	Optimal surveillance interval unknown; event rates low, limiting statistical power for risk stratification	Prospective registries defining normal post-implantation imaging findings and late event rates
Intermediate-concern phenotype (small PDL, CCTA-only patency, low-grade HAT, borderline geometry without embolic events)	Individualized repeat imaging interval determined by morphology, device type, temporal change, and clinical context; avoid reflexive antithrombotic escalation for CCTA-only findings	No randomized imaging-triggered management evidence; natural history of low-grade HAT and CCTA-only patency incompletely defined; risk of overtreatment in bleeding-prone population	Prospective multimodal imaging cohorts with standardized grading and clinical-outcome linkage
Higher-concern phenotype (definite DRT, high-grade HAT, large/persistent PDL, leak + DRT, adverse geometry + surface abnormality)	Closer surveillance and repeat imaging to confirm DRT resolution; temporary or resumed OAC when bleeding risk permits; consider percutaneous PDL closure in carefully selected cases	No randomized trial comparing DRT treatment strategies; PDL closure limited to case series; outcome-linked HAT and PDL risk thresholds not validated	Randomized trials of DRT management; prospective PDL closure registries with standardized definitions and independent outcome adjudication
Combined phenotype (e.g., small PDL + high-grade HAT + prior stroke)	Prompt multidisciplinary discussion; consideration of intensified surveillance and individualized antithrombotic reassessment	Virtually no evidence for combined-phenotype management; risk may be synergistic or additive but this remains speculative	Prospective multimodal imaging cohorts with prespecified combined-phenotype definitions and adjudicated outcomes

CCTA, cardiac computed tomography angiography; DRT, device-related thrombus; HAT, hypoattenuated thickening; LAA, left atrial appendage; MDT, multidisciplinary team; OAC, oral anticoagulation; PDL, peri-device leak.

This framework is provisional. All management decisions should be individualized and made through multidisciplinary discussion, particularly when imaging findings are discordant, bleeding risk is high, or evidence is uncertain.

## 7 Future directions and conclusions

The next phase of post-LAAO imaging research should move from descriptive surveillance toward outcome-linked validation. Current imaging can identify DRT, HAT, PDL, residual LAA patency, and device-appendage mismatch with increasing sensitivity. The clinical meaning of many findings, however, remains incompletely defined. Without standardized definitions and validated thresholds, more sensitive imaging may increase uncertainty rather than improve patient outcomes.

The priorities are practical. Image acquisition and interpretation should be standardized, including consistent TEE multiplane interrogation, leak measurement methods, and criteria for DRT adjudication. CCTA protocols require standardized gating, contrast timing, delayed-phase imaging, and attenuation-based criteria (15, 16). Reporting should move beyond binary labels: device surface abnormalities should be described according to morphology, grade, location, device type, and temporal evolution; residual communication should distinguish morphologic, hemodynamic, and clinical-risk leak (12, 14).

Prospective validation against clinical outcomes represents the most important next step. Existing evidence remains vulnerable to confounding by antithrombotic therapy, device generation, surveillance timing, and patient selection. Future studies should determine whether specific imaging findings, alone or in combination, predict stroke, systemic embolism, bleeding, DRT resolution, or need for reintervention. Only such studies can determine whether imaging-triggered treatment changes improve outcomes rather than simply increasing treatment burden (28, 70).

Artificial intelligence and computational modeling may support future workflows by improving segmentation, measurement reproducibility, and flow-based risk assessment (57, 58, 64–68, 73). These tools should be considered hypothesis-generating until externally validated against clinical outcomes.

In conclusion, post-LAAO imaging is entering a risk-interpretation phase. DRT is uncommon but potentially consequential. HAT is a heterogeneous, CCTA-defined surface phenotype whose clinical significance depends on grade, morphology, device type, and temporal evolution. PDL and residual LAA patency are common but variably actionable. Device-appendage geometry provides the structural context for understanding why these abnormalities occur. Available quantitative and longitudinal data support graded rather than binary interpretation of HAT. At present, this three-domain framework remains provisional and requires prospective validation. Improved standardization and outcome-linked thresholds will be necessary before imaging-defined residual risk can be meaningfully incorporated into clinical decision pathways.
